# Low density lipoprotein receptor gene *Ava *II polymorphism and serum lipid levels in the Guangxi Bai Ku Yao and Han populations

**DOI:** 10.1186/1476-511X-10-34

**Published:** 2011-02-23

**Authors:** Xing-Jiang Long, Rui-Xing Yin, Ke-La Li, Wan-Ying Liu, Lin Zhang, Xiao-Li Cao, Lin Miao, Dong-Feng Wu, Lynn Htet Htet Aung, Xi-Jiang Hu

**Affiliations:** 1 Department of Cardiology, Institute of Cardiovascular Diseases, the First Affiliated Hospital, 22 Shuangyong Road, Nanning 530021, Guangxi, People's Republic of China

## Abstract

**Background:**

Several common genetic polymorphisms in the low density lipoprotein receptor (LDL-R) gene have associated with modifications of serum total cholesterol (TC) and low density lipoprotein cholesterol (LDL-C) levels, but the results are not consistent in different populations. Bai Ku Yao is a special subgroup of the Yao minority in China. The present study was undertaken to detect the association of LDL-R gene *Ava *Ⅱ polymorphism and serum lipid levels in the Guangxi Bai Ku Yao and Han populations.

**Methods:**

A total of 1024 subjects of Bai Ku Yao and 792 participants of Han Chinese were randomly selected from our previous stratified randomized cluster samples. Genotyping of the LDL-R gene *Ava *Ⅱ polymorphism was performed by polymerase chain reaction and restriction fragment length polymorphism combined with gel electrophoresis, and then confirmed by direct sequencing.

**Results:**

The levels of serum TC, high density lipoprotein cholesterol (HDL-C), LDL-C, apolipoprotein (Apo) A1 and the ratio of ApoA1 to ApoB were lower in Bai Ku Yao than in Han (*P *< 0.01 for all). The frequency of A^- ^and A^+ ^alleles was 65.5% and 34.5% in Bai Ku Yao, and 80.7% and 19.3% in Han (*P *< 0.001); respectively. The frequency of A^-^A^-^, A^-^A^+ ^and A^+^A^+ ^genotypes was 42.6%, 45.9% and 11.5% in Bai Ku Yao, and 64.9%, 31.6% and 3.5% in Han (*P *< 0.001); respectively. There was also significant difference in the genotypic frequencies between males and females in Bai Ku Yao (*P *<0.05), and in the genotypic and allelic frequencies between normal LDL-C (≤ 3.20 mmol/L) and high LDL-C (>3.20 mmol/L) subgroups in Bai Ku Yao (*P *< 0.05 for each) and between males and females in Han (*P *< 0.05 for each). The levels of LDL-C in males and TC and HDL-C in females were different among the three genotypes (*P *< 0.05 for all) in Bai Ku Yao, whereas the levels of HDL-C in males and HDL-C and ApoA1 in females were different among the three genotypes (*P *< 0.05-0.001) in Han. The subjects with A^+^A^+ ^genotype had higher serum LDL-C, TC, HDL-C or ApoA1 levels than the subjects with A^-^A^+ ^and A^-^A^- ^genotypes. Spearman rank correlation analysis revealed that the levels of LDL-C in Bai Ku Yao and HDL-C in Han were correlated with genotypes (*P *< 0.05 and *P *< 0.01; respectively).

**Conclusions:**

The association of LDL-R gene *Ava *Ⅱ polymorphism and serum lipid levels is different between the Bai Ku Yao and Han populations. The discrepancy might partly result from different LDL-R gene *Ava *Ⅱ polymorphism or LDL-R gene-enviromental interactions.

## Introduction

Dyslipidemia including high levels of plasma or serum total cholesterol (TC) [1,2], triglycerides (TGs) [3,4], low density lipoprotein cholesterol (LDL-C) [5,6] and apolipoprotein (Apo) B [7,8], and low levels of ApoA1 [7,8] and high density lipoprotein cholesterol (HDL-C) [9,10], is a growing healthcare problem and risk factor for common diseases such as atherosclerosis, coronary heart diseases (CHD) and hypertension [11]. It has been recognized that dyslipidemia is a multifactorial disease involving environmental [12-14] and genetic factors [15-21] and their interactions [22,23]. Family and twin studies have shown that genetic polymorphisms could account for 40-60% of the interindividual variation in plasma lipid concentrations [24-26].

The low density lipoprotein receptor (LDL-R), a transmembrane cellular protein, plays a crucial role in the receptor-mediated pathway of lipoprotein metabolism [27,28]. The LDL-R modulates plasma levels of LDL-C by regulating the uptake of LDL particles by the liver and delivers cholesterol to the adrenal glands and gonads for steroid hormone synthesis and to the liver for bile acid synthesis [28,29]. Mutations in the LDL-R gene that disturb the normal functions of the LDL-R protein can cause familial hypercholesterolemia (FH), which is associated with elevated total and LDL-C and premature CHD [30,31]. FH, however, accounts for only about 5% of patients with CHD, and the contribution of genes to CHD in the remaining 95% of cases is still unknown [32]. Common single nucleotide polymorphisms (SNPs) in genes involved in lipid metabolism are potentially important genetic markers in affecting normal variation in the plasma or serum lipid profiles and thus determining susceptibility or resistance to CHD in the general population. Since the LDL-R gene plays an important role in cholesterol homeostasis [28], it is also possible that genetic variations in this gene may exist that have only a small effect on the function of the receptor. If such variations are common, they may make an important contribution in determining plasma lipid levels in the general population. The human LDL-R gene consists of 18 exons and 17 introns with the length of approximately 45 kb, and is mapped to chromosome 19p13.2. [33,34]. More than 12 restriction fragment length polymorphisms (RFLPs) within and near the LDL-R gene have been reported [35,36]. A common *Ava *Ⅱ polymorphism has been found to be associate with variation of plasma or serum lipid levels in some studies [37-47] but not in others [48-50].

There are fifty-six ethnic groups in China. Han is the largest group and Yao is the eleventh largest minority among the 55 minority groups according to the population size. Bai Ku Yao (White-trouser Yao), a special and isolated subgroup of the Yao minority, is named so because all the men wear white knee-length knickerbockers. The population size is about 30000. Because of isolation from the other ethnic groups, the special customs and cultures including their clothing, intra-ethnic marriages, dietary habits, and corn wine and rum intakes are still completely preserved to the present day. In several previous epidemiological studies, we found that the serum lipid parameters were lower in Bai Ku Yao than in Han Chinese from the same region [12-14]. This ethnic difference in serum lipid profiles is still not well known. We postulated that there may be significant differences in some lipid metabolism-related genetic polymorphisms between the two ethnic groups. Therefore, the aim of the present study was to detect the association of LDL-R gene *Ava *Ⅱ polymorphism and serum lipid levels in the Guangxi Bai Ku Yao and Han populations.

## Materials and methods

### Study population

A total of 1024 subjects of Bai Ku Yao who reside in Lihu and Baxu villages in Nandan County, Guangxi Zhuang Autonomous Region, People's Republic of China were randomly selected from our previous stratified randomized cluster samples [12-14]. The ages of the subjects ranged from 15 to 80 years, with an average age of 36.01 ± 18.16 years. There were 468 males (45.70%) and 556 females (54.30%). All subjects were rural agricultural workers. The subjects accounted for 3.41% of total Bai Ku Yao population. During the same period, a total of 792 people of Han Chinese who reside in the same villages were also randomly selected from our previous stratified randomized cluster samples [12-14]. The mean age of the subjects was 35.67 ± 18.63 years (range 15 to 80). There were 330 men (41.67%) and 462 women (58.33%). All of them were also rural agricultural workers. All study subjects were essentially healthy and had no evidence of any chronic illness, including hepatic, renal, or thyroid. The participants with a history of heart attack or myocardial infarction, stroke, congestive heart failure, diabetes or fasting blood glucose ≥ 7.0 mmol/L determined by glucose meter have been excluded. The participants were not taking medications known to affect serum lipid levels (lipid-lowering drugs such as statins or fibrates, beta-blockers, diuretics, or hormones). The present study was approved by the Ethics Committee of the First Affiliated Hospital, Guangxi Medical University. Informed consent was obtained from all subjects after they received a full explanation of the study.

### Epidemiological survey

The survey was carried out using internationally standardized methods, following a common protocol [51]. Information on demographics, socioeconomic status, and lifestyle factors was collected with standardized questionnaires. The alcohol information included questions about the number of liangs (about 50 g) of rice wine, corn wine, rum, beer, or liquor consumed during the preceding 12 months. Alcohol consumption was categorized into groups of grams of alcohol per day: ≤ 25 and ≥ 25. Smoking status was categorized into groups of cigarettes per day: ≤ 20 and ≥ 20. At the physical examination, body height, weight, and waist circumference were measured. Sitting blood pressure was measured three times with the use of a mercury sphygmomanometer after the subjects had a 5-minute rest, and the average of the three measurements was used for the level of blood pressure. Systolic blood pressure was determined by the first Korotkoff sound, and diastolic blood pressure by the fifth Korotkoff sound. Body weight, to the nearest 50 grams, was measured using a portable balance scale. Subjects were weighed without shoes and in a minimum of clothing. Height was measured, to the nearest 0.5 cm, using a portable steel measuring device. From these two measurements body mass index (BMI, kg/m^2^) was calculated. Waist circumference was measured with a nonstretchable measuring tape, at the level of the smallest area of the waist, to the nearest 0.1 cm.

### Lipid analysis

A venous blood sample of 8 mL was obtained from all subjects between 8 and 11 AM, after at least 12 hours of fasting, from a forearm vein after venous occlusion for few seconds in a sitting position. A part of the sample (3 mL) was collected into glass tubes and allowed to clot at room temperature, and used to determine serum lipid levels. Another part of the sample (5 mL) was transferred to tubes with anticoagulate solution (4.80 g/L citric acid, 14.70 g/L glucose, and 13.20 g/L tri-sodium citrate) and used to extract DNA. Immediately following clotting serum was separated by centrifugation for 15 minutes at 3000 rpm. The levels of TC, TG, HDL-C, and LDL-C in samples were determined by enzymatic methods with commercially available kits, Tcho-1, TG-LH (RANDOX Laboratories Ltd., Ardmore, Diamond Road, Crumlin Co. Antrim, United Kingdom, BT29 4QY), Cholestest N HDL, and Cholestest LDL (Daiichi Pure Chemicals Co., Ltd., Tokyo, Japan); respectively. Serum ApoA1 and ApoB levels were detected by the immunoturbidimetric immunoassay using a commercial kit (RANDOX Laboratories Ltd.). All determinations were performed with an autoanalyzer (Type 7170A; Hitachi Ltd., Tokyo, Japan) in the Clinical Science Experiment Center of the First Affiliated Hospital, Guangxi Medical University [12-14].

### DNA amplification and genotyping

Genomic DNA was extracted from peripheral blood leukocytes using the phenol-chloroform method [15-21]. The extracted DNA was stored at 4°C until analysis. Genotyping of the LDL-R gene *Ava *Ⅱ polymorphism was performed by polymerase chain reaction and restriction fragment length polymorphism (PCR-RFLP) [32,48]. PCR amplification was carried out with forward primer 5'-GTCATCTTCCTTGCTGCCTGTTTAG-3' and reverse primer 5'-GTTTCCACAAGGAGGTTTCAAGGTT-3' (Sangon, Shanghai, People's Republic of China). Each amplification reaction was performed in a total volume of 25 mL, containing 3 μL of genomic DNA, 1.0 μL of each primer (10 μmol/L), 7.5 μL of ddH_2_O, and 12.5 μL 2 × *Taq *PCR MasterMix (constituent: 0.1 U *Taq *polymerase/μL, 500 μM dNTP each and PCR buffer). After initial denaturizing at 94°C for 3 min, the reaction mixture was subjected to 30 cycles of 30 s denaturation at 94°C, 30 s annealing at 55°C and extension 60 s at 72°C, followed by a final 5 min extension at 72°C. After electrophoresis on a 2.5% agarose gel with 0.5 μg/mL ethidium bromide, the amplification products were visualized under ultraviolet light. Then 10 U of *Ava *Ⅱ enzyme was added directly to the PCR products (8 μL) and digested at 37°C overnight. After restriction enzyme digestion of the amplified DNA, the genotypes were identified by electrophoresis on 2.5% agarose gels and visualized with ethidium-bromide staining ultraviolet illumination. The genotypes were scored by an experienced reader blinded to epidemiological data and serum lipid levels. Six samples (A^-^A^-^, A^-^A^+ ^and A^+^A^+ ^genotypes in two; respectively) detected by the PCR-RFLP were also confirmed by direct sequencing. The PCR products were purified by low melting point gel electrophoresis and phenol extraction, and then the DNA sequences were analyzed in Shanghai Sangon Biological Engineering Technology & Services Co., Ltd., People's Republic of China.

### Diagnostic criteria

The normal values of serum TC, TG, HDL-C, LDL-C, ApoA1, ApoB levels, and the ratio of ApoA1 to ApoB in our Clinical Science Experiment Center were 3.10-5.17, 0.56-1.70, 0.91-1.81, 2.70-3.20 mmol/L, 1.00-1.78, 0.63-1.14 g/L, and 1.00-2.50; respectively. The individuals with TC >5.17 mmol/L and/or TG >1.70 mmol/L were defined as hyperlipidemic [12-14]. Hypertension was diagnosed according to the criteria of 1999 World Health Organization-International Society of Hypertension Guidelines for the management of hypertension [52,53]. The diagnostic criteria of overweight and obesity were according to the Cooperative Meta-analysis Group of China Obesity Task Force. Normal weight, overweight and obesity were defined as a BMI < 24, 24-28, and >28 kg/m^2^; respectively [54].

### Statistical analyses

Epidemiological data were recorded on a pre-designed form and managed with Excel software. All statistical analyses were done with the statistical software package SPSS 13.0 (SPSS Inc., Chicago, Illinois). Quantitative variables were expressed as mean ± standard deviation (SD), and qualitative variables as percentages. Allele frequency was determined via direct counting, and the standard goodness-of-fit test was used to test the Hardy-Weinberg equilibrium. Differences in genotype distribution between the groups were obtained using the chi-square test. The difference in general characteristics between Bai Ku Yao and Han was tested by the Student's unpaired *t*-test. The association of serum lipid variables with genotypes was tested by analysis of covariance (ANCOVA). Sex, age, BMI, blood pressure, alcohol intake, cigarette smoking were adjusted for the statistical analysis. In order to confirm the association of serum lipid parameters and genotypes (A^-^A^- ^= 1, A^-^A^+ ^= 2 and A^+^A^+ ^= 3), Spearman rank correlation analysis was also performed in the combined population of Bai Ku Yao and Han, Bai Ku Yao, and Han; respectively. A *P *value of less than 0.05 was considered statistically significant.

## Results

### General characteristics and serum lipid levels

Table 1 gives the general characteristics and serum lipid levels between the Bai Ku Yao and Han populations. The levels of body height, weight, TC, HDL-C, LDL-C, ApoA1 and the ratio of ApoA1 to ApoB were lower in Bai Ku Yao than in Han (*P *< 0.01 for all), whereas the percentages of subjects who consumed alcohol or smoked cigarettes were higher in Bai Ku Yao than in Han (*P *< 0.01 for each). There was no significant difference in the levels of BMI, systolic blood pressure, diastolic blood pressure, pulse pressure, TG, ApoB, age structure, and the ratio of male to female between the two ethnic groups (*P *>0.05 for all).

**Table 1:** The general characteristics and serum lipid levels between the Bai Ku Yao and Han populations

Parameter	Bai Ku Yao	Han Chinese	*t *(*x*^2^)	*P*
Number	1024	792	-	-
Male/female	468/556	330/462	2.954	0.085
Age (years)	36.01 ± 18.16	35.67 ± 18.63	0.275	0.783
Height (cm)	151.84 ± 7.13	156.30 ± 7.82	-12.670	0.000
Weight (kg)	49.98 ± 7.19	53.26 ± 10.30	-7.982	0.000
Body mass index (kg/m^2^)	21.64 ± 2.47	21.75 ± 3.64	-0.766	0.444
Waist circumference (cm)	70.80 ± 7.76	71.09 ± 10.27	-0.486	0.627
Systolic blood pressure (mmHg)	116.94 ± 16.68	117.36 ± 17.36	-0.361	0.718
Diastolic blood pressure (mmHg)	74.06 ± 9.41	73.77 ± 10.57	0.444	0.657
Pulse pressure (mmHg)	42.89 ± 12.09	43.59 ± 11.89	-0.878	0.380
Cigarette smoking [n (%)]				
Nonsmoker	786 (76.8)	636 (80.3)		
<20 cigarettes/day	120 (11.7)	58 (7.3)		
≥ 20 cigarettes/day	118 (11.5)	98 (12.4)	9.791	0.007
Alcohol consumption [n (%)]				
Nondrinker	688 (67.2)	646 (81.6)		
<25 g/day	216 (21.1)	106 (13.4)		
≥ 25 g/day	120 (11.7)	40 (5.1)	50.079	0.000
Total cholesterol (mmol/L)	4.21 ± 0.96	4.51 ± 0.99	-4.624	0.000
Triglyceride (mmol/L)	1.23 ± 0.80	1.25 ± 0.91	-0.358	0.721
HDL-C (mmol/L)	1.58 ± 0.42	1.74 ± 0.49	-5.350	0.000
LDL-C (mmol/L)	2.48 ± 0.74	2.63 ± 0.79	-2.991	0.003
Apolipoprotein (apo) AI (g/L)	1.24 ± 0.32	1.34 ± 0.36	-4.708	0.000
ApoB (g/L)	0.81 ± 0.24	0.79 ± 0.21	1.562	0.119
ApoAI/ApoB	1.63 ± 0.65	1.78 ± 0.60	-3.561	0.000

HDL-C, highdensity lipoprotein cholesterol; LDL-C, low-density lipoprotein cholesterol

### Results of electrophoresis and genotyping

**Figure 1:**
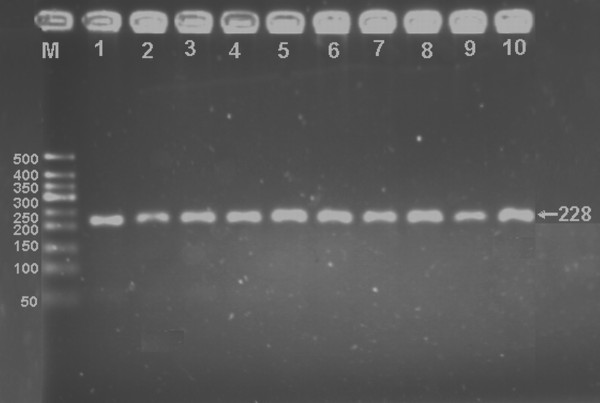
**Electrophoresis of PCR products of the samples**. Lane M, 50 bp marker ladder; lanes 1-10, samples. The 228 bp bands are the PCR products.

**Figure 2:**
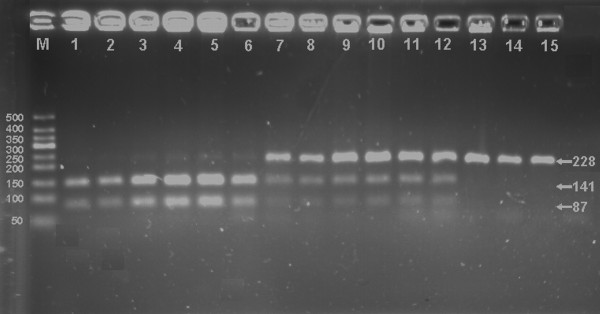
**Genotyping of the LDL-R gene *Ava *Ⅱ polymorphism**. Lane M, 50 bp marker ladder; lanes 1-6, A^+^A^+ ^genotype (141- and 87-bp); lanes 7-12, A^-^A^+ ^genotype (228-, 141- and 87-bp); and lanes 13-15, A^-^A^- ^genotype (228-bp).

After the genomic DNA of the samples was amplified by PCR and imaged by 2.5% agarose gel electrophoresis, the PCR products of 228 bp nucleotide sequences could be found in all samples (Figure 1). The genotypes identified were named according to the presence or absence of the enzyme restriction sites. The presence of the cutting site indicates the A^+ ^allele, while its absence indicates the A^- ^allele (cannot be cut). Thus, A^+^A^+ ^genotypes were homozygotes for the presence of the site (141- and 87-bp), A^-^A^+ ^genotypes were heterozygotes for the absence and presence of the site (228-, 141- and 87-bp), and A^-^A^- ^genotypes were homozygotes for the absence of the site (228-bp; Figure 2); respectively. The genotypic distribution was consistent with the Hardy-Weinberg equilibrium.

### Genotypic and allelic frequencies

The genotypic and allelic frequencies of LDL-R gene *Ava *Ⅱ polymorphism in Bai Ku Yao and Han Chinese are shown in Table 2. The frequency of A^- ^and A^+ ^alleles was 65.5% and 34.5% in Bai Ku Yao, and 80.7% and 19.3% in Han (*P *< 0.001); respectively. The frequency of A^-^A^-^, A^-^A^+ ^and A^+^A^+ ^genotypes was 42.6%, 45.9% and 11.5% in Bai Ku Yao, and 64.9%, 31.6% and 3.5% in Han (*P *< 0.001); respectively. There was also significant difference in the genotypic frequencies between males and females (*P *< 0.05) and in the genotypic and allelic frequencies between normal LDL-C (≤ 3.20 mmol/L) and high LDL-C (>3.20 mmol/L; *P *< 0.05 for each) subgroups in Bai Ku Yao, and in the genotypic and allelic frequencies between males and females in Han (*P *< 0.05 for each).

**Table 2:** Comparison of the genotypic and allelic frequencies of LDL-R gene *Ava *Ⅱ polymorphism in Bai Ku Yao and Han Chinese [n (%)]

Group	n	Genotype			Allele	
			
		**A**^-^**A**^-^	**A**^-^**A^+^**	A^+^A^+^	**A**^-^	A^+^
Bai Ku Yao	1024	436 (42.6)	470 (45.9)	118 (11.5)	1342 (65.5)	706 (34.5)
Han Chinese	792	514 (64.9)	250 (31.6)	28 (3.5)	1278 (80.7)	306 (19.3)
*x*^2^	-	101.118			102.054	
*P*	-	0.000			0.000	
Bai Ku Yao						
Male	468	218(46.6)	190(40.6)	60(12.8)	626(66.9)	310(33.1)
Female	556	220(39.6)	278(50.0)	58(10.4)	718(64.6)	394(35.4)
*x*^2^	-	9.095			1.204	
*P*	-	0.011			0.272	
Normal TC	882	368 (41.7)	420 (47.6)	94 (10.7)	1156 (65.5)	608 (34.5)
High TC	142	68 (47.9)	50 (35.2)	24 (16.9)	186 (65.5)	98 (34.5)
*x*^2^	-	9.332			0.000	
*P*	-	0.009			0.990	
Normal LDL-C	902	392 (43.5)	414 (45.9)	96 (10.6)	1198 (66.4)	606 (33.6)
High LDL-C	122	44 (36.1)	56 (45.9)	22 (18.0)	144 (59.0)	100 (41.0)
*x*^2^	-	6.472			5.199	
*P*	-	0.039			0.023	
Han Chinese						
Male	330	230 (69.7)	92 (27.9)	8 (2.4)	552 (83.6)	108 (16.4)
Female	462	284 (61.5)	158 (34.2)	20 (4.3)	726 (78.6)	198 (21.4)
*x*^2^	-	6.418			6.337	
*P*	-	0.040			0.012	
Normal TC	622	412 (66.2)	190 (30.6)	20 (3.2)	1014 (81.5)	230 (18.5)
High TC	170	102 (60.0)	60 (35.3)	8 (4.7)	264 (77.6)	76 (22.4)
*x*^2^	-	2.593			2.558	
*P*	-	0.274			0.109	
Normal LDL-C	636	414 (65.1)	202 (31.8)	20 (3.1)	1030 (81.0)	242 (19.0)
High LDL-C	156	100 (64.1)	48 (30.8)	8 (5.1)	248 (79.5)	64 (20.5)
*x*^2^	-	1.452			0.356	
*P*	-	0.484			0.550	

TC, total cholesterol; LDL-C, low-density lipoprotein cholesterol; Normal TC, TC ≤ 5.17 mmol/L, High TC, TC >5.17 mmol/L; Normal LDL-C, LDL-C ≤ 3.20 mmol/L, High LDL-C, LDL-C >3.20 mmol/L

### Results of sequencing

**Figure 3:**
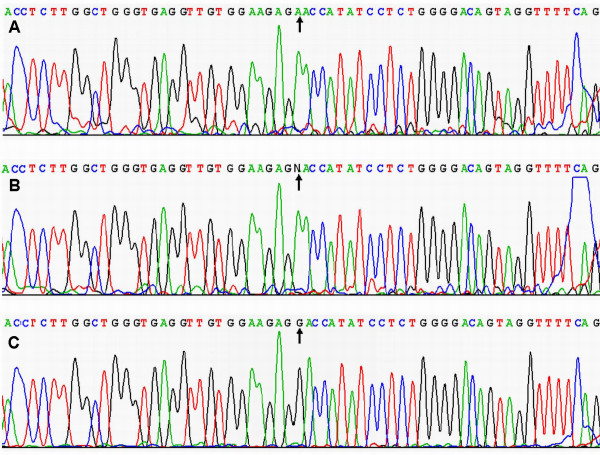
**A part of the nucleotide sequence of LDL-R gene *Ava ***Ⅱ **polymorphism**. (A) A^-^A^- ^genotype; (B) A^-^A^+ ^genotype; (C) A^+^A^+ ^genotype.

The results were shown as A^-^A^-^, A^-^A^+ ^and A^+^A^+ ^genotypes by PCR-RFLP, the A^-^A^-^, A^-^A^+ ^and A^+^A^+ ^genotypes were also confirmed by sequencing (Figure 3); respectively.

### Genotypes and serum lipid levels

As shown in Table 3, the levels of TC and LDL-C in Bai Ku Yao were significant differences among the three genotypes (*P *< 0.05 and *P *< 0.01; respectively). The subjects with A^+^A^+ ^genotype had higher serum TC levels than the subjects with A^-^A^+ ^genotypes, and higher serum LDL-C levels than the subjects with A^-^A^+ ^and A^-^A^- ^genotypes. When the associations of genotypes and serum lipid parameters were analyzed according to sex, the levels of LDL-C in males and TC and HDL-C in females were significant differences among the three genotypes (*P *< 0.05 for all). The subjects with A^+^A^+ ^genotype had higher serum TC, LDL-C or HDL-C levels than the subjects with A^-^A^+ ^and A^-^A^- ^genotypes.

**Table 3:** Comparison of serum lipid levels among the genotypes of LDL-R gene *Ava *Ⅱ polymorphism in Bai Ku Yao and Han Chinese

Genotype	n	TC (mmol/L)	TG (mmol/L)	HDL-C (mmol/L)	LDL-C (mmol/L)	ApoAI (g/L)	ApoB (g/L)
Bai Ku Yao							
A^-^A^-^	436	4.23 ± 0.98	1.26 ± 0.82	1.58 ± 0.42	2.44 ± 0.69	1.24 ± 0.33	0.82 ± 0.23
A^-^A^+^	470	4.14 ± 0.81	1.19 ± 0.79	1.56 ± 0.40	2.45 ± 0.65	1.22 ± 0.31	0.81 ± 0.24
A^+^A^+^	118	4.38 ± 0.86^c^	1.27 ± 0.73	1.66 ± 0.49	2.71 ± 1.12^bd^	1.28 ± 0.33	0.86 ± 0.24
*F*	-	3.673	1.050	2.676	6.792	1.724	2.122
*P*	-	0.026	0.350	0.069	0.001	0.179	0.120
Male							
A^-^A^-^	218	4.17 ± 1.16	1.36 ± 1.00	1.57 ± 0.45	2.34 ± 0.80	1.25 ± 0.38	0.75 ± 0.27
A^-^A^+^	190	4.05 ± 0.90	1.28 ± 1.00	1.56 ± 0.50	2.32 ± 0.70	1.23 ± 0.40	0.75 ± 0.23
A^+^A^+^	60	4.23 ± 0.86	1.30 ± 0.79	1.62 ± 0.53	2.69 ± 1.45^bc^	1.27 ± 0.36	0.82 ± 0.29
*F*	-	1.030	0.355	0.359	4.443	0.287	1.936
*P*	-	0.358	0.123	0.699	0.012	0.751	0.146
Female							
A^-^A^-^	220	4.28 ± 0.77	1.17 ± 0.58	1.59 ± 0.38	2.55 ± 0.53	1.23 ± 0.28	0.86 ± 0.18
A^-^A^+^	278	4.20 ± 0.75	1.13 ± 0.62	1.56 ± 0.31	2.53 ± 0.60	1.21 ± 0.23	0.85 ± 0.23
A^+^A^+^	58	4.53 ± 0.84^ad^	1.23 ± 0.69	1.70 ± 0.45^ac^	2.73 ± 0.64	1.30 ± 0.31	0.90 ± 0.17
*F*	-	4.494	0.732	3.746	2.917	2.901	1.420
*P*	-	0.012	0.482	0.024	0.055	0.056	0.243
Han Chinese							
A^-^A^-^	514	4.49 ± 1.02	1.23 ± 0.87	1.70 ± 0.47	2.63 ± 0.82	1.34 ± 0.34	0.79 ± 0.21
A^-^A^+^	250	4.56 ± 0.94	1.31 ± 0.99	1.78 ± 0.50	2.63 ± 0.76	1.35 ± 0.38	0.79 ± 0.20
A^+^A^+^	28	4.44 ± 1.08	1.17 ± 1.08	2.14 ± 0.54^bd^	2.60 ± 0.77	1.40 ± 0.45	0.79 ± 0.23
*F*	-	0.486	0.759	12.262	0.018	0.407	0.004
*P*	-	0.615	0.469	0.000	0.982	0.666	0.996
Male							
A^-^A^-^	230	4.45 ± 0.94	1.35 ± 1.13	1.62 ± 0.48	2.64 ± 0.75	1.30 ± 0.37	0.80 ± 0.21
A^-^A^+^	92	4.45 ± 0.93	1.50 ± 1.28	1.73 ± 0.44	2.54 ± 0.82	1.33 ± 0.39	0.76 ± 0.20
A^+^A^+^	8	3.80 ± 1.45	1.56 ± 1.78	1.77 ± 0.46	2.17 ± 0.92	1.02 ± 0.21	0.66 ± 0.21
*F*	-	1.823	0.598	2.059	1.818	2.549	2.725
*P*	-	0.163	0.550	0.129	0.164	0.080	0.067
Female							
A^-^A^-^	284	4.53 ± 1.09	1.13 ± 0.56	1.76 ± 0.45	2.62 ± 0.87	1.36 ± 0.32	0.78 ± 0.22
A^-^A^+^	158	4.62 ± 0.95	1.20 ± 0.76	1.82 ± 0.54	2.69 ± 0.72	1.37 ± 0.38	0.81 ± 0.20
A^+^A^+^	20	4.79 ± 0.66	0.95 ± 0.38	2.35 ± 0.49	2.83 ± 0.61	1.56 ± 0.42^ac^	0.87 ± 0.70
*F*	-	0.857	1.640	13.965	0.874	3.123	1.658
*P*	-	0.425	0.195	0.000	0.418	0.045	0.192

TC, total cholesterol; TG, triglycerides; HDL-C, high-density lipoprotein cholesterol; LDL-C, low-density lipoprotein cholesterol; ApoAI, apolipoprotein AI; ApoB, apolipoprotein B; ^a^*P *< 0.05 and ^b^*P *< 0.01 in comparison with A^-^A^- ^genotype of same group; ^c^*P *< 0.05 and ^d^*P *< 0.01 in comparison with A^-^A^+ ^genotype of same group.

The levels of HDL-C in Han Chinese were significant differences among the three genotypes (*P *< 0.001). The subjects with A^+^A^+ ^genotype had higher serum HDL-C levels than the subjects with A^-^A^+ ^and A^-^A^- ^genotypes. When the assocoations of genotypes and serum lipid parameters were analyzed according to sex, the levels of HDL-C in males and HDL-C and ApoA1 in females were significant differences among the three genotypes (*P *< 0.05-0.001). The subjects with A^+^A^+ ^genotype had higher serum HDL-C and ApoA1 levels than the subjects with A^-^A^+ ^and A^-^A^- ^genotypes.

### Risk factors for the lipid parameters

Spearman rank correlation analysis showed that serum LDL-C levels in Bai Ku Yao were correlated with genotypes (*P *< 0.05), whereas serum HDL-C levels in Han Chinese were associated with genotypes (*P *< 0.01; Table 4).

**Table 4:** The results of Spearman rank correlation analysis between serum lipid parameters and the genotypes of LDL-R gene *Ava *Ⅱ polymorphism

Regression coefficient	Bai Ku Yao and Han	Bai Ku Yao	Han Chinese
*r*_TC_	0.016	0.028	0.011
*r*_TG_	0.009	0.008	0.013
*r*_HDL-C_	0.062	0.018	0.116**
*r*_LDL-C_	0.053	0.092*	0.014
*r*_ApoA1_	0.026	0.020	0.028
*r*_ApoB_	0.022	0.017	0.034

*r*, regression coefficient; TC, total cholesterol; TG, triglyceride; HDL-C, high-density lipoprotein cholesterol; LDL-C, low-density lipoprotein cholesterol; ApoA1, apolipoprotein A1; ApoB, apolipoprotein B; *P < 0.05 and **P < 0.01.

## Discussion

The present study showed that the levels of serum TC, HDL-C, LDL-C, ApoA1 and the ratio of ApoA1 to ApoB were lower in Bai Ku Yao than in Han. There was no significant difference in serum TG and ApoB levels between the two ethnic groups. These findings are consistent with those of our previous epidemiological studies [12-14]. It is well known that dyslipidemia is a complex trait caused by multiple environmental and genetic factors and their interactions. Differences in serum lipid profiles between the two ethnic groups might mainly result from different dietary patterns, lifestyle factors as well as genetic background. Bai Ku Yao is an isolated subgroup of the Yao minority in China. There are about 30000 people of total Bai Ku Yao population who reside in two villages, Lihu and Baxu, Nandan County. Their ancestors began their migration from Hunan and Guizhou Province about Song Dynasty (a ruling dynasty in China between 960 and 1279). Both Lihu and Baxu villages are typical infertile mountain area, usually it was called 30 percent soil with 70 percent rock. Their income mostly comes from planting corn and paddy. Thus, corn was the staple food and rice, soy, buckwheat, sweet potato, and pumpkin products were the subsidiary foods in Bai Ku Yao. Approximately 90% of the beverages were corn wine and rum. The alcohol content is about 15% (v/v). They are also accustomed to drink hempseed soup and eat hempseed products. Several experimental and clinical studies have demonstrated that the Hempseed products and Hempseed oil can decrease serum TC, TG and LDL-C levels [55-57]. In contrast, rice was the staple food and corn, broomcorn, potato, and taro products were the subsidiary foods in Han. About 90% of the beverage was rice wine. The content of alcohol is about 30% (v/v). The staple and subsidiary foods are more favorable for serum lipid profiles in Bai Ku Yao than in Han [58-62]. In addition, strict intra-ethnic marriages have been performed in this ethnic subgroup from time immemorial. Namely, only both Bai Ku Yao man and woman can intermarriage, and can not intermarry with the other subgroup of Yao minority or other ethnic groups. Therefore, this unique ethnic subgroup with a homogenous environmental and genetic background is a useful tool for genetic study.

Several previous studies have shown that the genotypic and allelic distribution of LDL-R gene *Ava *Ⅱ polymorphism was different in diverse populations. Ahn *et al. *[32] investigated the effect of LDL-R gene *Ava *Ⅱ polymorphism on plasma lipid profiles in 385 normolipidemic Hispanics and 543 non-Hispanic whites from the San Luis Valley, Colorado. They showed that the frequency of A^+ ^allele was higher in Hispanics than in non-Hispanic whites (56% vs. 43%; *P <*0.001). Salazar *et al. *[38] reported that the frequency of A^+^A^+ ^genotype was higher in 170 white unrelated individuals presenting a lipid profile with high risk for CHD compared to that of the 130 controls (32% vs. 16%) from São Paulo City, Brazil. Salazar *et al. *[40] also found that the frequency of A^+ ^allele was higher in 50 patients with FH than in 130 normal controls (58% vs. 45%; *P *= 0.036). The FH patients showed a greater frequency of A^+^A^+ ^homozygous genotypes when compared to the normolipidemic controls (*P *= 0.0292). In addtion, several studies also showed that the frequency of A^+ ^allele was higher in patients with acute myocardial infarction [45], phlegm-dampness constitution [47], gallstone disease [48], but was lower in patients with atherosclerotic cerebral infarction [50] as compared with the corresponding normal controls; respectively. Liu *et al. *[43] found that the frequency of A^+ ^allele in males but not in females was higher in hyperlipidemic (24.51%) and margin lipid patients (25.00%) than in normal controls (11.11%; *P *< 0.05 for each). Several studies, however, showed that there was no significant difference in the frequency of A^+ ^allele between the CHD and control groups [44,46], and between male and female Han children [49]. In the present study, we showed that the frequency of A^+ ^allele and A^-^A^+ ^and A^+^A^+ ^genotypes was higher in Bai Ku Yao than in Han. There was also significant difference in the genotypic frequencies between males and females in Bai Ku Yao, and in the genotypic and allelic frequencies between normal LDL-C (≤ 3.20 mmol/L) and high LDL-C (>3.20 mmol/L) subgroups in Bai Ku Yao and between males and females in Han. These results indicate that the prevalence of the A^+ ^allele variants of LDL-R gene *Ava *Ⅱ polymorphism may have a racial/ethnic specificity.

The association of LDL-R gene *Ava *Ⅱ polymorphism and plasma or serum lipid levels in humans are inconsistent in several previous studies. Ahn *et al. *[32] reported that the LDL-R gene *Ava *Ⅱ polymorphism revealed a gender-specific effect on plasma lipid profiles. The gender-specific effect was significant for HDL-C (*P *< 0.001) and TG (*P *= 0.001). Ethnicity affected only the levels of TG (*P *= 0.02). Significant variability among *Ava *II genotypes was observed for TC and LDL-C in non-Hispanic women (*P *= 0.0015 and *P *= 0.014) and TC in Hispanic women (*P *= 0.011) and was highly suggestive for LDL-C in Hispanic women (*P *= 0.057). For both non-Hispanic and Hispanic women the trends seen in the cholesterol levels were similar: low in the A^-^A^- ^genotype, intermediate in the A^-^A^+ ^genotype, and high in the A^+^A^+ ^genotype. Neither significant nor directional results were seen in men in either ethnic group. In non-Hispanic women the estimated average effect of the A^+ ^allele was to raise LDL-C by 4.95 mg/dL, and the average effect of the A^- ^allele was to lower LDL-C by 3.68 mg/dL; this variation explained 3.9% of the phenotypic variance in TC and 2.3% in LDL-C. In Hispanic women the estimated average effect of the A^+ ^allele was to increase LDL-C by 2.92 mg/dL, and the A^- ^allele lowered levels by 3.81 mg/dL; this variation explained 3.7% and 2% of the phenotypic variance in TC and LDL-C; respectively. Humphries *et al. *[37] showed that the individuals with one or two A^- ^alleles (absence of cutting site) had levels of LDL-C that were lower by 8.0% and 12.4%; respectively. Variation associated with this RFLP explained 4.2% of the sample variance. Pongrapeeporn *et al. *[41] also revealed that the mean LDL-C levels was slightly higher in the A^+^A^+ ^genotype than the A^-^A^+ ^and A^-^A^- ^genotypes in 54 normolipidemic Thai subjects. The difference was significant at the 5 per cent level although there were only three homozygotes with the A^+^A^+ ^genotype. The average effect of the A^+ ^allele was to increase LDL-C level by 6.75 mg/dl. A gene-dosage effect was not observed for this polymorphism. In addition, the subjects with the A^+^A^+ ^genotype also tended to have high serum TC and TG and low HDL-C levels. The LDL-R gene *Ava *Ⅱ polymorphism was also found to be associate with the variability of plasma or serum lipid levels in subjects presenting a lipid profile with high risk for CHD [38], in normal individuals from five Brazilian Indian tribes [39], in males with hypercholesterolemia [43], in cases with acute myocardial infarction [45], in patients with CHD [46], and in population with phlegm-dampness constitution [47]. Several studies, however, failed to find a significant association between the LDL-R gene *Ava *Ⅱ polymorphism and plasma or serum lipid levels in normal controls [48,50] or in Han children [49]. In the present study, we showed that the levels of TC and LDL-C in Bai Ku Yao were significant differences among the three genotypes. The subjects with A^+^A^+ ^genotype had higher serum TC levels than the subjects with A^-^A^+ ^genotypes, and higher serum LDL-C levels than the subjects with A^-^A^+ ^and A^-^A^- ^genotypes. When the statistical analyses were performed according to sex, the levels of LDL-C in males and TC and HDL-C in females were significant differences among the three genotypes. The subjects with A^+^A^+ ^genotype had higher serum LDL-C, TC or HDL-C levels than the subjects with A^-^A^+ ^and A^-^A^- ^genotypes. The levels of HDL-C in Han Chinese were significant differences among the three genotypes. The subjects with A^+^A^+ ^genotype had higher serum HDL-C levels than the subjects with A^-^A^+ ^and A^-^A^- ^genotypes. When the analyses were performed for each gender category separately, the levels of HDL-C in males and HDL-C and ApoA1 in females were significant differences among the three genotypes. The subjects with A^+^A^+ ^genotype had higher serum HDL-C and ApoA1 levels than the subjects with A^-^A^+ ^and A^-^A^- ^genotypes. Spearman rank correlation analysis showed that serum LDL-C levels in Bai Ku Yao were correlated with genotypes, whereas serum HDL-C levels in Han Chinese were associated with genotypes. The reason for this discrepancy between the two ethnic groups may relate to the difference in the LDL-R gene *Ava *Ⅱ polymorphism and/or LDL-R gene-enviromental interactions [44].

## Conclusion

The present study shows that the frequency of A^+ ^allele and A^-^A^+ ^and A^+^A^+ ^genotypes was higher in Bai Ku Yao than in Han. There was also significant difference in the genotypic frequencies between males and females and in the genotypic and allelic frequencies between normal LDL-C (≤ 3.20 mmol/L) and high LDL-C (>3.20 mmol/L) subgroups in Bai Ku Yao, and in the genotypic and allelic frequencies between males and females in Han. The levels of LDL-C in males and TC and HDL-C in females were different among the three genotypes in Bai Ku Yao, whereas the levels of HDL-C in males and HDL-C and ApoA1 in females were different among the three genotypes in Han. The A^+^A^+ ^homozygotes had higher serum LDL-C, TC, HDL-C or ApoA1 levels than the A^-^A^+ ^heterozygotes and A^-^A^- ^homozygotes. The levels of LDL-C in Bai Ku Yao and HDL-C in Han were correlated with genotypes. The difference in the association of LDL-R gene *Ava *Ⅱ polymorphism and serum lipid levels between the two ethnic groups might partly result from different LDL-R gene *Ava *Ⅱ polymorphism or LDL-R gene-enviromental interactions.

## Competing interests

The authors declare that they have no competing interests.

## Authors' contributions

XJL participated in the design, undertook genotyping, and helped to draft the manuscript. RXY conceived the study, participated in the design, carried out the epidemiological survey, collected the samples, and drafted the manuscript. KLL, WYL, LZ, XLC, LM, DFW, LHHA and XJH collaborated to the genotyping. All authors read and approved the final manuscript.
